# Development of Geraniol-Loaded Liposomal Nanoformulations against *Salmonella* Colonization in the Pig Gut

**DOI:** 10.1021/acs.jafc.2c00910

**Published:** 2022-06-02

**Authors:** Sotirios I. Ekonomou, Pooja Akshay Thanekar, Dimitrios A. Lamprou, Edward Weaver, Olena Doran, Alexandros Ch. Stratakos

**Affiliations:** † Faculty of Health and Applied Sciences (HAS), University of the West of, Coldharbour Ln, Bristol BS16 1QY, England; ‡ School of Pharmacy, Queen’s University Belfast, 97 Lisburn Road, Belfast BT9 7BL, UK

**Keywords:** antivirulence, gastrointestinal tract, *Salmonella*, liposomes, delivery, microfluidics

## Abstract

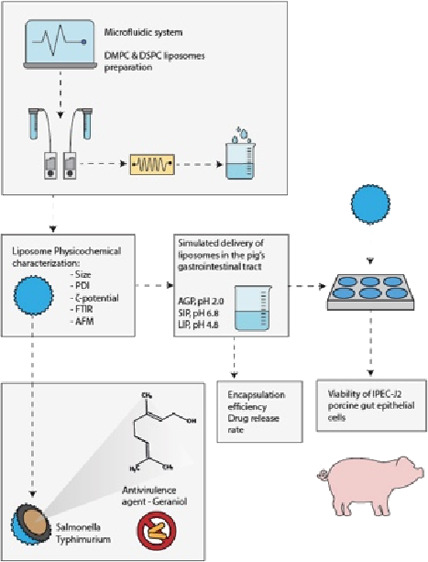

*Salmonella* is a global health threat, with pig production being one of the main sources of human salmonellosis. The current study investigated the antivirulence properties of geraniol for inhibiting the in vitro colonization of *Salmonella*. The minimum inhibitory (MIC) and bactericidal concentrations (MBC) of geraniol against *Salmonella typhimurium* followed by the sub-MIC of geraniol were determined. Results provided clear evidence that geraniol at 1/8 MIC can be used as an effective, non-toxic antivirulence compound to inhibit virulence factors (motility, adhesion, and invasiveness) affecting the colonization of *S. typhimurium* on IPEC-J2 cells. Additionally, the findings signified that microfluidics is an emerging technology suitable for the preparation of stable liposomes with a small size (<200 nm) and high encapsulation efficiency (EE) of up to 92.53%, which can act as effective carriers of geraniol into the pig gastrointestinal tract (GIT), targeting *Salmonella*, preventing colonization, and thus increasing the safety of the food supply chain.

## Introduction

*Salmonella* is a ubiquitous foodborne enteric pathogen causing more than 93 million cases of salmonellosis and 155,000 deaths annually worldwide.^1^ According to the latest report of the European Food Safety Authority (EFSA), in 2021, salmonellosis was the second most frequent cause of gastrointestinal (GI) infection in humans in the European Union (EU)^2^ as well as in the United States of America (USA).^3^ Human infection with *Salmonella* has been mainly associated with the direct consumption of contaminated raw or undercooked poultry and pork products.^4^*Salmonella* commonly adheres to the pig intestinal epithelial cells, and infection can occur at different production stages.^5^ For this reason, *Salmonella* can be transmitted through the livestock’s oral–fecal route at the environment, the farm, the slaughterhouse, and the food processing plant, where it can survive and cross-contaminate the equipment or the final food products, causing human infections.^4^*S. typhimurium* is one of the main well-described serotypes that are associated with the pig production chain and commonly multidrug-resistant, causing severe cases in humans.^1,6−8^

Excessive use of antibiotics to cure diseases as well as control and prevent pathogens in modern animal production plants has led to the emergence of highly resistant pathogenic bacteria, including *Salmonella* strains.^8^ It is commonly accepted that there is a dose-dependent relationship between the use of antibiotics and the development of resistance in bacteria. In most cases, the animals receive the antibiotics as feed or water additives. The most resistant zoonotic bacteria constitute a major public health risk due to their transmission to humans through the food chain. In the last two decades, an increased prevalence of multidrug-resistant *Salmonella* strains has been observed to clinically important antimicrobial agents such as fluoroquinolones and third-generation cephalosporins used to treat severe *Salmonella* infections in humans.^14^

The increased antimicrobial resistance associated with the intensive use of antibiotics has led to searching for new animal production alternatives. As a result, a broad range of natural essential oils (EOs) and their major compounds have been used in the last decades as a substitute to antibiotics in animal feed and to help tackle the global threat of antibiotic resistance. EOs are secondary metabolites obtained from plants, spices, herbs, and fruits and consist of a complex mixture of low-molecular weight compounds.^9−11^ EOs can be used as antivirulence agents, providing an alternative strategy to inhibit the virulence factors of pathogenic bacteria that facilitate human disease. The antivirulence approach aims at suppressing the expression of virulence factors that are crucial for bacterial pathogenicity.^12^ This approach does not affect the survival or growth of the targeted microorganism; thus, it would not change the overall selective pressures, meaning that is less likely to lead to the development of resistance.^13^

Geraniol (*trans*-3,7-dimethyl-2,6-oktadien-1-ol) is a monoterpenic alcohol and naturally occurs as a major compound in many EOs, such as geranium or rose oils and has many applications in a variety of products, including food.^14^ It is a non-toxic, hydrophobic compound classified in the category of generally recognized as safe (GRAS) by the US Food and Drug Administration and the European Food Security Agency.^15^ In addition, it is an effective compound that has been highlighted for its antimicrobial properties against numerous pathogens.^16,17^ However, there are only a limited number of studies on the use of geraniol as an antivirulence agent against bacterial pathogens.^18,19^

If geraniol is to be applied as an additive in pig feed or water, the initial administrated levels are unlikely to be reached in the gastrointestinal tract (GIT) since it can be diluted in the gut by binding to the feed’s protein and lipid constituents.^18^ Other reasons limiting the successful application of hydrophobic compounds as feed/water additives are their low solubility in aqueous media in parallel with rapid hydrolysis.^22^ The use of liposomes has attracted wide attention recently to encapsulate hydrophobic compounds and improve their solubility, stability, and bioactivity in the animal’s GIT.^20,21^ Liposomes are spherical bilayer vesicles of varying sizes with an aqueous inner compartment that can be self-assembled naturally or in vitro with natural or synthetic food-grade lipids accepted as GRAS.^25^ Various methods are employed to prepare liposomes, and most recently, microfluidics has gained popularity. The application of microfluidics overcomes the problems of conventional methods for liposome fabrication resulting in more stable and uniform formulations with high repeatability, showing the potential for large scale production.^23,24^ Microfluidics also offers other advantages, such as the fabrication of liposomes by a simple continuous flow process through the channels of a microfluidic chip in the sub-nanoliter scale and the use of a small volume of reagents in significantly less time.^25^

The aim of this study was to investigate the antivirulence properties of geraniol against *S. typhimurium* with focus on the inhibition of motility, adherence, and invasiveness on IPEC-J2 porcine intestinal epithelial cells. We also aimed to encapsulate geraniol in liposomal formulations acting as carriers to increase its stability and solubility in the pig GIT. A comprehensive characterization of the geraniol loaded liposomes was also performed to determine their stability, physicochemical characteristics, and in vitro release kinetics. The potential toxicity of free and encapsulated geraniol on IPEC-J2 cells was also examined.

## Materials and Methods

### Bacterial Strain and Growth Conditions

*Salmonella enterica* serovar Typhimurium (*S. typhimurium*, ATCC 14028) was used from a frozen stock stored at −80 °C in Cryoinstant vials with porous beads (Microbank, Pro-Lab Diagnostics, UK). A single bead was transferred aseptically in sterile Mueller Hinton broth (MHB; Oxoid, UK) to activate the culture and incubated overnight at 37 °C. From the overnight culture, a 100 μL inoculum was transferred in 10 mL MHB and incubated at 37 °C for 24 h. To prepare the working culture, the cells were centrifuged at 6500*g* at 4 °C for 10 min, washed twice with phosphate-buffered saline (pH 7.4; PBS; Oxoid, UK), and finally diluted in 10 mL of MHB to an appropriate bacterial population before use.

### Determination of the Minimum Inhibitory Concentration and Minimum Bactericidal Concentration of Geraniol

The broth macrodilution method was used to determine the MIC of geraniol (Merck, UK) at concentrations of 0.05, 0.10, 0.50, 1.00, 2.00, and 5.00% (v/v) (0.5–50 mg/mL). Portions of stock geraniol were added in 9.9 mL of MHB before inoculation to obtain target concentrations (v/v) following the addition of 100 μL of the *S. typhimurium* inoculum to reach a final population of approximately 10^6^ CFU/tube. Test tubes inoculated with *S. typhimurium* were allowed to incubate at 37 °C for 24 h. The MIC was defined at the lowest concentration of geraniol, where no turbidity was identified, meaning the growth of *S. typhimurium* was inhibited after incubation.

MBC was determined by adding 100 μL of suspension from the tubes where no turbidity was identified on tryptone soy agar (TSA; Oxoid, UK) plates and incubated at 37 °C for 24 h. The MBC was reported as the minimum concentration of geraniol at which there was more than 99.9% reduction of the initial inoculum as determined by plate counts on TSA. Negative controls without geraniol or bacterial suspension were also included to detect any possible cross-contamination. All experiments were conducted in triplicate.

### Determination of Subinhibitory Concentrations (Sub-MIC) of Geraniol

The broth microdilution method for antibacterial testing was carried out as recommended by the CLSI protocol^26^ with some modifications. To identify the sub-MIC of geraniol against *S. typhimurium*, 150 μL of MHB was dispensed in the wells of a sterile 96-well plate. In the first well of each row, 150 μL of geraniol were added to achieve the MIC value identified previously, and then two-fold serial dilutions were made. Next, 150 μL of bacterial suspension was added to each well to achieve a final population of 1.5 × 10^6^ CFU/mL. Negative controls without geraniol or *S. typhimurium* suspension were also included in the last two wells to detect possible cross-contamination. The 96-well plates were incubated at 37 °C for 18 h aerobically without shaking using the Omega plate reader (FLUOstar, Omega, BMG Labtech, UK). The growth kinetics of *S. typhimurium* were monitored by optical density measurements at 600 nm (OD_600_).

### Motility Determination

The effect of geraniol on *S. typhimurium* swarming motility was determined according to Inamuco et al.^27^ A single colony grown on TSA was stabbed into semisolid medium agar with or without geraniol at concentrations ranging from 0 to 1/8 MIC. Bacterial motility was assessed after incubation at 37 °C for 24 h.

### Cell Culture and Adhesion and Invasion to IPEC-J2 Porcine Gut Epithelial Cells

The intestinal porcine epithelial cells (IPEC-J2) were grown in Dulbecco’s modified Eagle’s medium (DMEM)–high-glucose (Sigma-Aldrich, UK) medium supplemented with 10% fetal bovine serum (FBS; Sigma-Aldrich, UK) at 37 °C and 5% CO_2_ in a humidified incubator. For infection and viability experiments, cells were grown to confluent monolayers in 7 days. Adhesion and invasion assays were performed according to Sima et al.^28^ The adhesion and invasion assays took place in the presence of geraniol at 1/8, 1/16, and 1/32 MIC in DMEM. Plate-grown *S. typhimurium* was harvested, washed, and resuspended in culture medium. Bacteria were added to the culture plates to give a multiplicity of infection of 100. Culture plates were centrifuged at 250*g* for 5 min and incubated for 3 h at 37 °C. All assays were conducted independently three times.

### Preparation of the Liposomal Formulations

The phosphatidylcholine (PC) liposomes were prepared using a LineUp Push-Pull pressure-controlled microfluidic system (Fluigent, Paris, FR). The 14:0 PC (DMPC):1,2-dimyristoyl-*sn*-glycero-3-phosphocholine and 18:0 PC (DSPC): 1,2-dimyristoyl-*sn*-glycero-3-phosphocholine lipids were purchased from Avanti Polar Lipids (Alabaster, AL). The microfluidic setup included a reservoir containing the aqueous solution of sterile PBS (pH 7.4), while the second one contained the solvent solution [99.8% pure ethanol (Merck, UK)] with a ratio of 2:1 lipids:cholesterol and geraniol at 1/8 MIC. A flow rate sensor (Fluigent, Paris, FR) allowed continuous monitoring of the microfluidic setup’s flow rate. The pressurized reservoirs’ fluids are injected through two separate inlet channels to allow mixing into a T-shaped microfluidic chip with a zigzag micromixing pattern (Darwin Microfluidics, Paris, FR). All liposomal formulations were prepared using the optimal combination of lipids:cholesterol ratio of 2:1,^29^ at a total flow ratio (TFR) of 1 mL/mL and a flow rate ratio (FRR) of 3:1 aqueous:solvent solution using the Fluigent SDK software (Fluigent, Paris, FR). The above mentioned liposomal formulation parameters were selected based on previous encapsulation and stability studies by our group^30^ in order to obtain an appropriate stability profile for PC liposomes.

### Investigation of the Physicochemical Characteristics of Liposomes

#### Determination of the Particle Size, Polydispersity Index, and ζ Potential

The stability test for the 14:0 PC (DMPC) and 18:0 PC (DSPC) with the encapsulated geraniol was performed for 4 weeks. The impact of the storage temperature on the liposomes’ stability was evaluated at 4, 20, and 39 °C. The 4 and 20 °C temperatures were chosen as they are common refrigeration and room temperatures. In both temperatures, a wide variety of biological, pharmacological, and agricultural products that include liposomal formulations can be stored over a long period and their stability in the final product is a prerequisite. The 39 °C storage temperature was chosen as it is the temperature encountered in pig GIT. Samples were collected the first day of the liposome formulation and every 7 days and were measured for their size, PDI, and ζ-potential. Ten microliters of the liposomal suspension was collected and added to 990 μL of PBS (1:100 dilution) and filtered through a 20 μm Millipore filter (Merck, Darmstadt, DE). The final sample was measured by dynamic light scattering (DLS) on a Zetasizer Nano-ZS (Malvern Instruments Ltd., UK). For the mean particle’s size and PDI measurements, a 1 mL disposable cuvette (Merck, UK) was used, while the Folded Capillary Zeta Cell (Malvern Panalytical, DE) was used for the determination of ζ potential. A fixed scattering angle of 137° was used to measure all the physicochemical characteristics of liposomes.

#### Fourier Transform Infrared Spectroscopy

Characterization of the DMPC and DSPC liposomes performed using an attenuated total reflection (ATR)–FTIR spectrometer (Nicolet 50, Thermo Fisher Scientific, UK), with built-in ATR. Analysis was performed to confirm the presence of all compounds within the formulation. All liposomal formulations were centrifuged at 14800 rpm at 25 °C and the supernatant was removed before analysis.

Scans were performed over a wave range of 4000–600 cm^–1^, over 128 scans at a resolution of 4 cm^–1^ and an interval of 1 cm^–1^. Background absorption was subtracted from the analysis.

#### Atomic Force Microscopy

A TT-2 AFM (AFM Workshop, US) was used to perform the AFM analysis and assist with the visualization and further characterization of the liposomal formulations. A volume of 10 μL from each liposomal formulation was diluted in 1800 μL of PBS. Fifteen microliters of this dilution was then pipetted onto a freshly cleaved mica surface (1.5 cm × 1.5 cm; G250–2 mica sheets 1″ × 1″ × 0.006″, Agar Scientific Ltd., Essex, UK). The samples were dried for 30 min and then imaged under ambient conditions. Ω cm antimony-doped Si probes (frequency = 167 kHz) were used to image the samples at a scan rate of 0.6 Hz and 512 × 512-pixel resolution over an area of 5 μm.

### Encapsulation Efficiency of Liposomes

The geraniol-loaded liposome suspension of 1 mL was added in a sterile Eppendorf tube and centrifuged twice at 11,000*g* for 20 min. After centrifugation, the pellets were washed with PBS (pH 7.4) and resuspended in PBS using the same initial volume before use. All samples collected were analyzed for geraniol concentration using a UV–Vis spectrophotometer (GENESYS 150, Thermo Scientific, UK). The geraniol standard solution was analyzed over the UV wavelength range of 200–400 nm, and the maximum absorption wavelength of geraniol was 208 nm. The linear relationship between the concentration of geraniol (*x*) and the UV absorbance (*y*) was created according to the calibration curve of spectrophotometry and was used to calculate the EE of the liposomes. The equation used to give the final concentration (%) of the encapsulated compound was
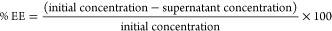
1

### *In Vitro* Drug Release of Liposomes under Simulated Gastrointestinal Conditions

To determine the release rate of encapsulated geraniol, a three-step protocol simulating the three different stages of the GIT of pigs was used. First, an aliquot of 1 mL of DMPC and DSPC liposomal geraniol-loaded formulations was added separately in the cellulose tubing membranes with an average flat width of 10 mm and 14,000 molecular weight cut-off (MWCO; Sigma, Darmstadt, DE). The cellulose tubing membrane was boiled for 30 min in distilled water and rinsed thoroughly before use. The liposomes were transferred in the dialysis tubing membrane, and both sides were closed using dialysis tubing closures (Sigma-Aldrich, Darmstadt, DE). The enclosed liposomes in the dialysis tubing membranes were added separately in sodium citrate buffer solution with pH values of 2.0, 6.8, and 4.8 for 2, 4, and 18 h at 39 °C, simulating the acid-gastric (AGP), small intestine (SIP), and large intestine phase (LIP), respectively. Finally, a 500 μL aliquot was extracted at specific time point intervals from the immersion medium, which was replaced with a new buffer solution pre-equilibrated at 39 °C to determine the dilution parameters. The concentration of free geraniol in the buffer solution was determined spectrophotometrically at 208 nm. The release rate (%) of geraniol during digestion was defined by eq 2:
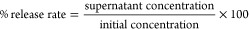
2

### Effect of Geraniol-Loaded Liposomes on the Viability of IPEC-J2 Porcine Gut Epithelial Cells

The effect on cell viability was determined by the 3-[4,5-dimethylthiazol-2-yl]-2,5-diphenyl tetrazolium bromide (MTT) assay (Roche, Sigma-Aldrich, UK) according to Ford et al.^31^ The cells at a population of 2 × 10^5^ were cultured in a 96-well plate for 18 h to allow cell attachment at 37 °C in a 5% CO_2_ atmosphere. The culture medium was then replaced with 100 μL of fresh medium containing free geraniol and geraniol-loaded liposomes. Control wells contained only fresh DMEM medium. After a 3 h incubation period, the media containing geraniol were removed and the cells were washed once with 100 μL of the fresh medium and replenished with 100 μL of fresh medium. Cell survival was evaluated by adding 10 μL of the MTT reagent (0.5 mg MTT/ml) to each well and incubating for 3 h. Subsequently, the medium was removed, 100 μL of the solubilization solution was added to dissolve the MTT formazan, and the plate was left for incubation overnight at 37 °C in a 5% CO_2_ atmosphere. The quantity of the MTT formazan was measured on an automatic plate reader (FLUOstar Omega, BMG Labtech, UK) in absorbance at 570 nm.^28^ The viable cells convert MTT into a purple-colored formazan. Cells viability was expressed as a percentage of control. All measurements were done in triplicate.

### Statistical Analysis

All data were expressed as mean ± standard deviation (SD) and were carried out at least in triplicates. When required, the data were subjected to a one-way ANOVA followed by Tukey post hoc test using the IBM SPSS Statistics 22 software (SPSS Inc., US). For data that showed a normal distribution, Student’s *t* test was used to determine significance at a 5% level of significance.

## Results and Discussion

### MIC and MBC Determination of Geraniol

In the first section, the antimicrobial activity of free geraniol was determined against *S. typhimurium.* EOs contain a number of compounds, and many of these compounds like carvacrol^27,32^ and thymol^33,34^ have been extensively studied for their antimicrobial and antivirulence effects; however, there is limited research about the effects of geraniol used in the current study.

Geraniol at a concentration of 0.10% (v/v) was found to inhibit the growth of *S. typhimurium*, while a bactericidal effect was observed at the concentration of 0.50% (v/v) (data not shown). Other studies showed that *S. typhimurium* strains DT104^35^ and ATCC 6539^36^ exhibited an MIC of 0.025% (v/v), while the MBCs ranged between 0.037 and 0.050% (v/v), respectively. In addition, geraniol has been proven to be effective against a number of different food and clinical pathogenic isolates.^16,17^ The antimicrobial effect of EOs and their major compounds, such as geraniol, is due to their solubility in the phospholipid bilayer of the cell membrane, leading to increased permeability and loss of cellular components.^37^

### Sub-MIC Determination of Geraniol

Antivirulence strategy has emerged as an alternative to conventional antimicrobial drugs, which act by killing bacteria or inhibiting bacterial growth. Antivirulence compounds work by inhibiting the virulence factors of the target bacterium. An increased number of reports underlined the potential of EOs and their compounds at sub-MICs in inhibiting several virulence factors of pathogenic bacteria.^13^ The current study provides insights on the antivirulence effect of geraniol at sub-MICs that can be applied to reduce the pathogenicity of *Salmonella* by inhibiting specific virulence factors and thus exerting a significantly less selective pressure for the development of resistance.

**Figure 1:**
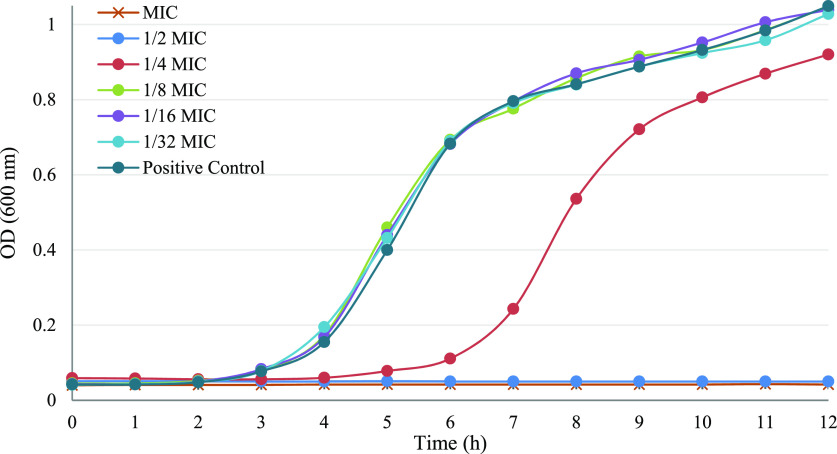
Growth kinetics of *S. typhimurium* in the presence of sub-MICs of geraniol at 37 °C. The values represent the mean optical density (OD) of three readings as mean ± SD; *n* = 3. Sub-MICs used are based on MIC values presented in the first section.

*S. typhimurium* was used as the target microorganism to evaluate the antimicrobial efficacy of geraniol and revealed typical sigmoidal kinetics and logarithmic growth after 12 h at 37 °C, as shown by the growth curves at the OD_600_ range (Figure 1). The action of geraniol at 1/2 MIC was apparent against *S. typhimurium*, while the bacterial growth was suppressed in the presence of 1/4 MIC. In contrast, growth at 1/8 MIC or lower concentrations appeared to be identical with the growth in the absence of geraniol (control). The sub-MICs of 1/8, 1/16, and 1/32 MIC that did not suppress the bacterial growth were selected and used in further experiments. The addition of EOs or their major compounds at sub-MIC levels in food and feed can be used to control and reduce the colonization of foodborne pathogens without the adverse effects of antibiotic resistance development. This is consistent with the results reported by Yuan and Yuk,^33^ who observed that thymol, carvacrol, and *trans*-cinnamaldehyde reduced *Escherichia coli* O157:H7 motility and biofilm-forming capacity. Stratakos et al.^32^ also investigated the in vitro effect of sub-inhibitory concentrations of carvacrol against different Shiga toxin-producing *E. coli* strains, showing that it can be potentially be applied as an antivirulence agent in food.

### Effect of Sub-MICs of Geraniol on the Motility, Adherence, and Invasiveness of *S. typhimurium* into IPEC-J2 Gut Epithelial Cells

Since *S. typhimurium* is an invasive, facultative intracellular pathogen that requires adhesion to cause pathogenesis to the host cells, the virulence factors of motility, adherence, and invasiveness of the pathogen in the presence of sub-MICs of geraniol were investigated.

**Figure 2:**
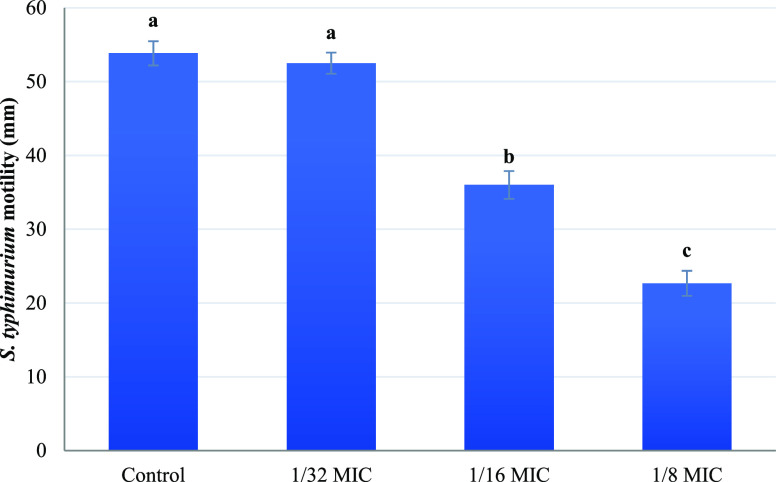
Effect of different concentrations of geraniol on the motility of *S. typhimurium* in soft agar plates. The bars represent the diameter of the area as mean ± SD; *n* = 3. Different lowercase letters indicate significant differences among treatments (*P* < 0.05).

**Figure 3:**
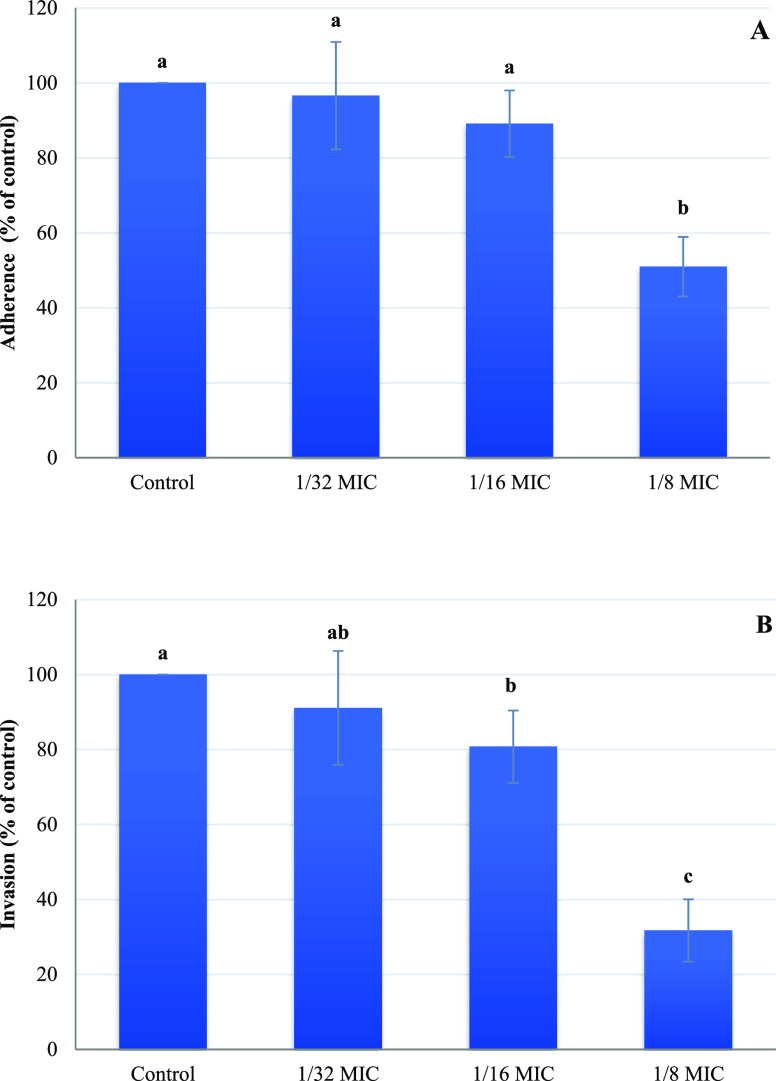
Adhesion (A) and invasion (B) of IPEC-J2 cells by *S. typhimurium*. Adhesion and invasion are presented as percentages relative to the control (set at 100%). The bars represent the means ± SD; *n* = 6. Different lowercase letters indicate significant differences among treatments (*P* < 0.05).

The 1/32 MIC had no significant effect on motility (Figure 2, *P* < 0.05). Similarly, no effect on the adhesion and invasion of *S. typhimurium* into IPEC-J2 gut epithelial cells was observed in the presence of 1/32 MIC (Figure 3A,B, *P* < 0.05), compared with the control samples. At 1/16 MIC of geraniol, *S. typhimurium* showed significantly reduced motility, whereas the highest motility reduction was observed when the bacterial cells were exposed to 1/8 MIC (Figure 2, *P* < 0.05). The decreased motility observed for *S. typhimurium* has also been described for *E. coli*(38) and *S. typhimurium* strain DT104 in the presence of carvacrol at concentrations that do not inhibit bacterial growth. Burt et al.^38^ reported that the reduced motility was caused due to the loss of *E. coli* flagellum, while Inamuco et al.^27^ presented that *S. typhimurium* (DT104) cells retained their flagellum in the presence of carvacrol and the reduced motility might be caused due to the loss of the flagellum’s functionality or disruption of bacterial quorum sensing.^39^ The results presented by Yuan and Yuk^33^ confirm this hypothesis where *E. coli* O157:H7 cells lost their motility when incubated in soft agar plates supplemented with sublethal concentrations of thymol and carvacrol due to the downregulation of the genes responsible for the biosynthesis of flagellar components. When the bacterial cells are subjected to stress conditions repress specific activities, such as motility to store energy and carry out other vital cellular functions.^40^ In a recent study, Zhang et al.^17^ reported that geraniol at various sub-MICs reduced not only the motility of *Pseudomonas fluorescens* and *Pectobacterium carotovorum* but also the production of the exopolysaccharide inhibiting this way the biofilm formation.

Bacterial motility plays a critical role in the adhesion and invasion of the pathogen, which can colonize the GIT.^41^ For this reason, it is expected that the reduced motility of *S. typhimurium* cells found will affect their capacity to adhere and invade into IPEC-J2 gut epithelial cells. In the current study, *S. typhimurium* in the presence of 1/16 MIC of geraniol did not show a statistically significant reduction in adhesion ability when compared with the 1/32 MIC and control (Figure 3A, *P* > 0.05), while a significantly lower invasion after treatment was revealed with 1/16 MIC compared with the control treatment (Figure 3B, *P* < 0.05). Although *S. typhimurium* adheres to the initial pathogenic niches on the cells’ membrane, infection at the host cell can only be achieved by invasion.^21^ Interestingly, the presence of sub-MIC (1/8 MIC) of geraniol resulted in a significant reduction of invasion (50.97%) and adhesion (31.72%) of *S. typhimurium* on the IPEC-J2 cells (Figure 3A,B; *P* < 0.05). Burt et al.^18^ reported that the natural compounds cinnamaldehyde and carvacrol, at sub-inhibitory concentrations, did not reduce the adherence of *S. typhimurium* on IPEC-J2 cells. However, carvacrol at sub-MIC was found to reduce the adherence of Shiga toxin-producing *E. coli* strains on HCT-8 cells.^32^ Moreover, other studies reported the reduced invasion of *S. typhimurium* on porcine epithelial cells and *Campylobacter jejuni* on INT-407 intestinal epithelial cells in the presence of 0.5 to 0.8 mM and 0.03 mg/mL of carvacrol, respectively.^27,42^ Based on our results, geraniol at sub-MICs could be effectively applied against *Salmonella* as an antivirulence agent. The mechanism by which geraniol and other antivirulence compounds affect adherence and invasiveness is not yet fully understood. However, it is known that their sub-inhibitory concentrations can regulate the genes’ transcription in various pathogens,^43^ including *Salmonella*.^44^

### Effect of Storage Temperatures to Physicochemical Characteristics of Geraniol-Loaded Liposomes

Liposomal formulations acting as delivery systems must be stable during storage for commercial applications. The microfluidic system used in the present study allows for better control of the particle size as well as increased stability of the final liposomes during preparation. The physicochemical characteristics of the formulated liposomes containing the encapsulated sub-MIC (1/8 MIC) of geraniol were evaluated using various techniques. The 1/8 MIC of geraniol was selected as it was the most effective one in inhibiting motility, adhesion, and invasiveness of *S. typhimurium* without affecting its growth or survival. The effect of three different storage temperatures at 4, 20 (room temperature), and 39 °C (pig GIT temperature) for 28 days was conducted to evaluate the stability of the liposomes with the encapsulated geraniol.

Particle composition may impact their stability during storage or within the GIT, altering their local molecular environment.^45^ Therefore, the size, PDI, and ζ potential of the liposomal formulations were investigated and presented in Tables 1 and 2. The initial size of the DMPC particles was 192.93 ± 2.04 nm with a PDI value of 0.225 ± 0.014 and a negative ζ potential charge of −21.23 ± 3.96 mV. The mean size of the DMPC particles on day 28 was 149.87 ± 3.43, 155.30 ± 4.48, and 176.50 ± 28.26 nm at 4, 20, and 39 °C without significant differences among the different storage temperatures (Table 1, *P* > 0.05). The DMPC liposomes after 28 days of storage at 4 and 20 °C revealed a statistically significant size decrease compared with day 0 (Table 1, *P* < 0.05). The shrinking observed for the DMPC liposomes might be due to osmosis, where geraniol is leaving the liposome core to a region of lower concentration (PBS). A similar phenomenon has been observed by Weaver et al.^30^ when the encapsulated bovine serum albumin left the liposome core causing shrinking of the liposomes during storage. Regarding the liposomes stored at 4 and 20 °C, the PDI remained low, showing that the storage temperature did not affect their size distribution (Table 1). A similar PDI of DMPC liposomes was obtained by Ballacchino et al.^46^ In most cases, ζ potential was in the range of −10 to −20 mV or even higher with some exceptions, such as for the liposome formulations at 39 °C after 14 days where a high decrease in size was observed showing lower stability (Table 1). A large positive or negative value of ζ potential (−25 to +25 mV) indicates good physical stability of nanostructures since the electrical charge of droplets is strong enough to suggest lower aggregation.^47^ Other factors such as material properties, the presence of surfactants, solution pH, and ionic strength may affect the physical stability of the obtained liposomes.^48^

**Table 1:** Main Physicochemical Characteristics of Geraniol-Loaded DMPC (18:0 PC) Liposomes at Various Storage Conditions for 28 Days^a^

day	0	7	14	21	28
size/nm					
4 °C	192.93 ± 2.04*^a,A^*	169.37 ± 4.21*^a,B^*	175.07 ± 3.75*^a,B^*	167.43 ± 3.65*^a,B^*	149.87 ± 3.43*^a,C^*
20 °C	192.93 ± 2.04^*a,A*^	161.53 ± 4.54^*a,B*^	174.80 ± 1.85^*a,C*^	162.50 ± 4.18^*a,B*^	155.30 ± 4.48^*a,B*^
39 °C	192.93 ± 2.04^*a,A*^	171.87 ± 11.22^*a,A*^	108.07 ± 3.86^*b,B*^	134.00 ± 5.54^*b,B*^	176.50 ± 28.26^*a,A*^
PDI					
4 °C	0.225 ± 0.014^*a,A*^	0.123 ± 0.016^*a,B*^	0.140 ± 0.018^*a,B*^	0.151 ± 0.011^*a,B*^	0.081 ± 0.007^*a,C*^
20 °C	0.225 ± 0.014^*a,A*^	0.102 ± 0.017^*a,B*^	0.172 ± 0.020^*a,C*^	0.153 ± 0.019^*a,C*^	0.065 ± 0.016^*a,B*^
39 °C	0.225 ± 0.014^*a,A*^	0.252 ± 0.052^*b,AB*^	0.340 ± 0.046^*b,ABC*^	0.305 ± 0.035^*b,BC*^	0.309 ± 0.049^*b,C*^
ζ potential (mV)					
4 °C	–21.23 ± 3.96^*a,A*^	–5.57 ± 0.70^*a,B*^	–14.66 ± 3.51^*a,A*^	–13.78 ± 8.68^*a,A*^	–14.44 ± 6.26^*ab,A*^
20 °C	–21.23 ± 3.96^*a,A*^	–8.20 ± 2.00^*a,B*^	–7.36 ± 2.93^*ab,B*^	–16.75 ± 6.37^*a,A*^	–8.53 ± 2.40^*a,B*^
39 °C	–21.23 ± 3.96^*a,A*^	–26.20 ± 1.85^*b,A*^	–5.06 ± 4.66^*b,B*^	–21.40 ± 5.57^*a,A*^	–22.73 ± 6.66^*b,A*^

a Values followed by different uppercase letters in each row are significantly different (*P* < 0.05). Values are presented as means ± SD (*n* = 3). Values followed by different lowercase letters in the same column are significantly different (*P* < 0.05). All values are presented as means ± SD (*n* = 3).

**Table 2:** Main Physicochemical Characteristics of Geraniol-Loaded DSPC (14:0 PC) Liposomes at Various Storage Conditions for 28 Days^a^

day	0	7	14	21	28
size (nm)					
4 °C	120.27 ± 2.55^*a,A*^	128.23 ± 2.22^*a,AB*^	132.00 ± 3.08^*a,B*^	155.97 ± 5.11 ^*a,C*^	157.20 ± 4.85^*a,C*^
20 °C	120.27 ± 2.55^*a,AB*^	114.70 ± 2.42^*b,A*^	119.47 ± 1.46^*b,AB*^	125.33 ± 2.90^*b,B*^	122.07 ± 3.06^*b,B*^
39 °C	120.27 ± 2.55^*a,A*^	124.83 ± 3.50^*a,AB*^	132.13 ± 3.72^*a,B*^	129.37 ± 2.08^*b,B*^	126.07 ± 2.76^*b,AB*^
PDI					
4 °C	0.116 ± 0.008^*a,A*^	0.093 ± 0.016^*a,A*^	0.069 ± 0.027^*a,A*^	0.084 ± 0.018^*a,A*^	0.086 ± 0.012^*a,A*^
20 °C	0.116 ± 0.008^*a,AB*^	0.123 ± 0.002^*b,AB*^	0.131 ± 0.011^*b,A*^	0.116 ± 0.007^*b,AB*^	0.107 ± 0.003^*a,B*^
39 °C	0.116 ± 0.008^*a,A*^	0.128 ± 0.012^*b,A*^	0.122 ± 0.018^*b,A*^	0.117 ± 0.002^*b,A*^	0.099 ± 0.012^*a,A*^
ζ potential (mV)					
4 °C	–5.50 ± 1.66^*a,A*^	–26.20 ± 9.01^*a,B*^	–19.80 ± 4.72^*a,AB*^	–21.63 ± 10.05^*a,B*^	–20.51 ± 5.93^*a,B*^
20 °C	–5.50 ± 1.66^*a,A*^	–13.93 ± 6.38^*ab,AB*^	–12.14 ± 2.62^*a,AB*^	–18.47 ± 6.43^*a,B*^	–14.84 ± 5.16^*a,B*^
39 °C	–5.50 ± 1.66^*a,A*^	–7.71 ± 1.13^*b,A*^	–13.63 ± 6.52^*a,AB*^	–17.90 ± 7.64^*a,B*^	–20.13 ± 4.28^*a,B*^

a Values followed by different uppercase letters in each row are significantly different (*P* < 0.05). Values are presented as means ± SD (*n* = 3). Values followed by different lowercase letters in the same column are significantly different (*P* < 0.05). All values are presented as means ± SD (*n* = 3).

DSPC liposomes loaded with geraniol presented an overall lower particle size of 120.27 ± 2.55 nm on day 0 compared with the DMPC formulations. Analogous size of DSPC liposomes was obtained by Guimarães Sá Correia et al.^24^ who used the same microfluidic method and FRR of 3:1 (aqueous:solvent solution). The lower (4 °C) temperature significantly affected the liposomes, leading to a larger size of 157.20 ± 4.85 nm at the end of the storage (Table 2). Interestingly, the liposomes stored at 20 and 39 °C showed high size stability without a significant increase even after 28 days (Table 2, *P* > 0.05). Temperature is another crucial aspect affecting the liposomes’ stability during storage.^48^ The PDI of the DSPC liposomes confirmed the above results, while in all cases, it remained close or lower than 0.1, a value indicating the monodisperse state. Other authors suggest that even a higher PDI value reaching up to 0.3 indicates homogenous and monodisperse formulations.^49^ Additionally, there was a significant increase in the ζ potential of the DSPC liposomes with the encapsulated geraniol after 28 days of storage. The liposomes at 4, 20, and 39 °C respectively presented ζ potentials of −20.51 ± 5.93, −14.84 ± 5.16, and – 20.13 ± 4.28 without significant differences among the tested temperatures (Table 2, *P* > 0.05). In some cases, fluctuations were observed in the surface charge of the liposomes. Although we cannot state conclusively the reason, we hypothesize that the fluctuations may be linked to the ionic changes in the medium, due to liposome degradation and geraniol release. Fluctuations at higher storage temperatures might also be due to the breakage of hydrogen bonds between the phospholipids, causing the rupture of the liposome membrane.^50^ Overall, the above results show the high potential for using DMPC and DSPC liposomes as efficient delivery vehicles to protect the encapsulated geraniol from degradation and improve the solubility into the pig GIT. Also, the liposomes produced in this study showed good stability for a 4 week storage period at 4, 20, and 39 °C, while similar results were obtained by Kastner et al.,^51^ who showed good liposome stability in terms of size over a period of up to 8 weeks at 4 and 25 °C.

### FTIR Measurements of Liposomes

**Figure 4:**
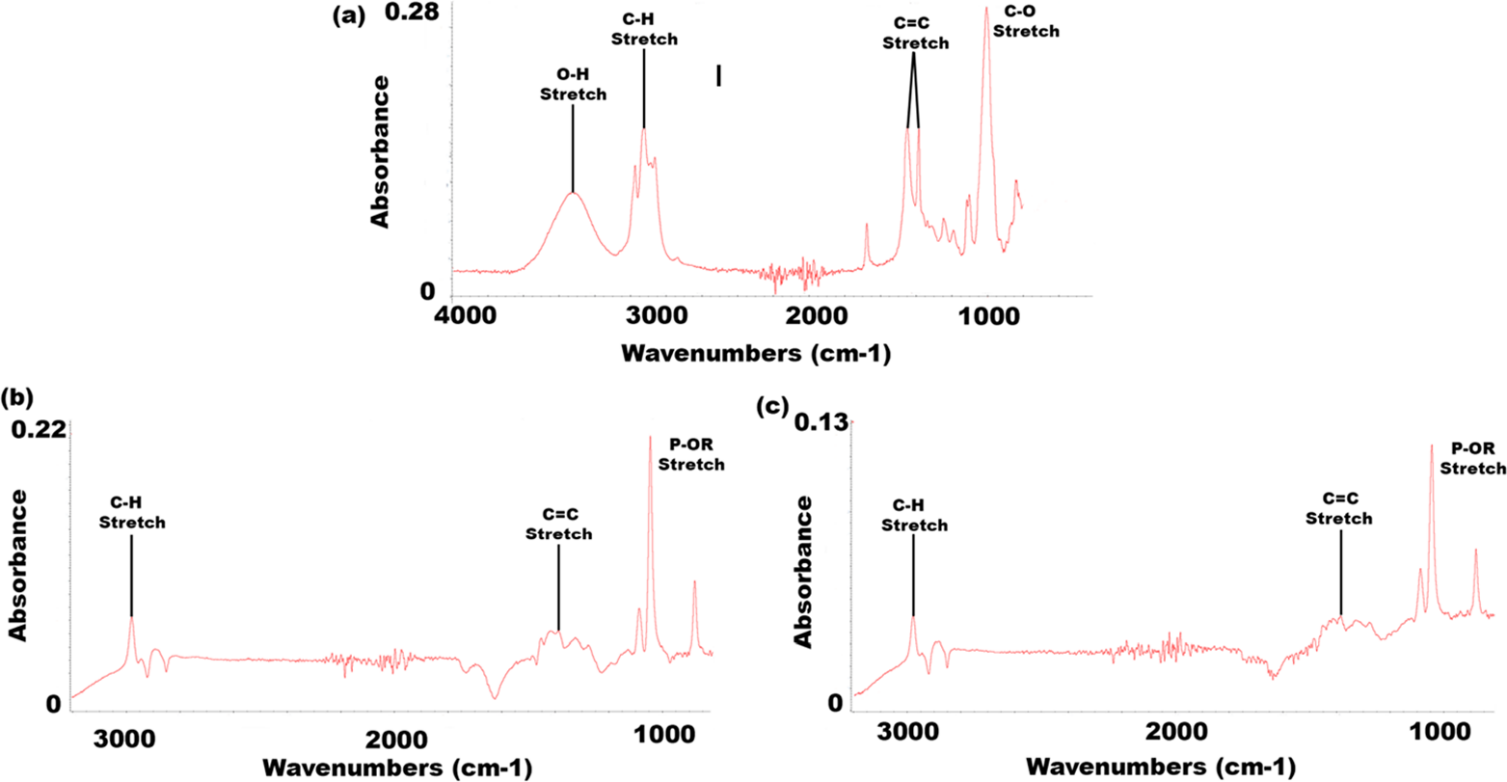
FTIR spectra of the investigated antimicrobial compound (a) geraniol and loaded liposomes with (b) DMPC and (c) DSPC.

FTIR spectroscopy was also used to characterize the DMPC and DSPC geraniol-loaded liposomes, as it has been proven to be a valuable method to provide insight by the resulting spectra that act as fingerprints for the tested compounds.^24,30^ The characteristic signals on FTIR spectra were marked in Figure 4.

The intensity bands of the liposomal formulations DSPC and DMPC are similar and are displayed by the spectra peaks at approximately 2900 cm^–1^, representing the C–H single-bond stretching (Figure 4b,c).^52^ The lack of signals responsible for the stretching vibrations of −OH groups in geraniol (3317.81 cm^–1^) on FTIR spectra^14^ (Figure 4a) and the disappearance of the signals connected with the stretching vibrations of C=O near 1700 cm^–1^ confirmed the encapsulation of geraniol during the preparation of the DMPC and DSPC liposomal formulations. The characteristic peak at approximately 1050 cm^–1^, which is in the focus of our investigation, carries information about the vibrations of P–OR phosphate head groups, proving the formulation of the liposomes (Figure 4b,c). In accordance with other studies, the main difference observed for the tested liposomes was the absorption intensity of the peaks at 1050 cm^–1^, which can be due to the presence of different lipid sizes.^24,30^

### AFM Imaging of Liposomes

**Figure 5:**
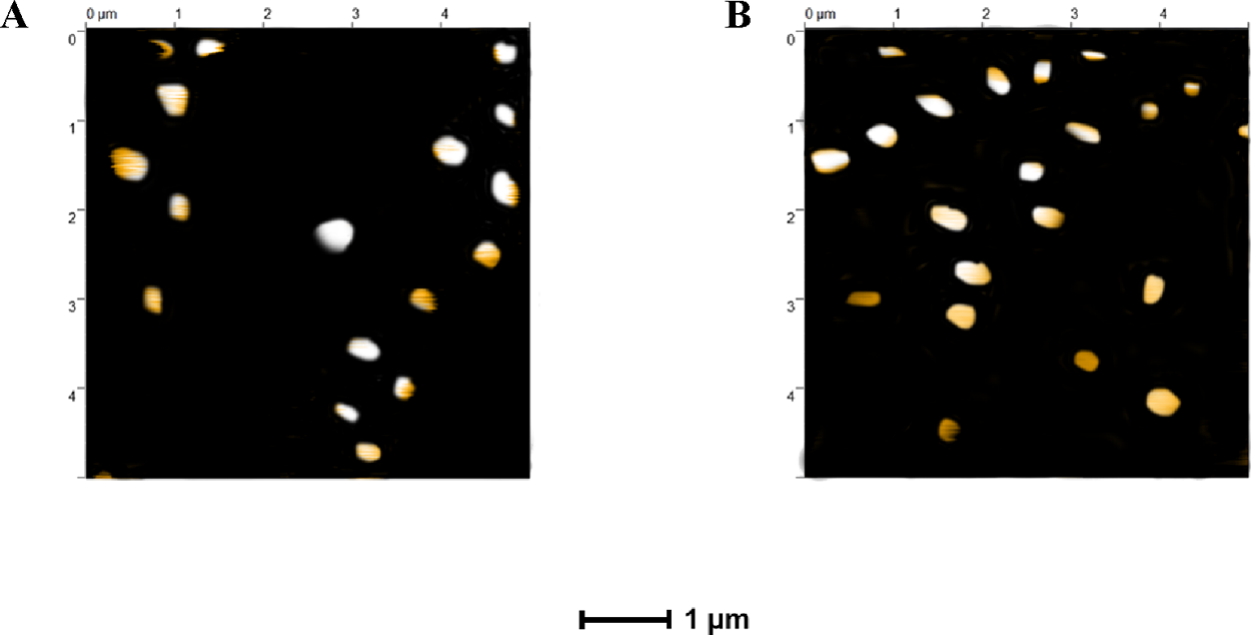
AFM images of (A) DMPC and (B) DSPC geraniol-loaded liposomes adsorbed on mica substrate.

AFM was used to visualize all liposomal formulations under dry conditions (Figure 5). The DSPC liposomes presented in Figure 5B revealed the smallest size with higher shape uniformity than the DMPC liposomes in Figure 5A. Compressed or flattened spheroidal shapes as observed in our DMPC and DSPC liposomes are frequently related to AFM technique limitations. It is generally accepted that many factors can modify the liposomes’ shape, such as the time of analysis, the type of liposomes, and the forces applied on them during the scanning procedure.^53^ Similar size and shape morphology with our results were observed for the DMPC-loaded liposomes when AFM imaging was performed.^30^ In agreement with Weaver et al.^30^ the size observed for both DMPC and DSPC liposomes (Figure 5A,B) using AFM imaging was larger compared with the size measured by DLS and ranged up to 300 nm, mainly due to the drying process that the liposomes undertook. AFM imaging can be used alternatively to the well-established transmission or scanning electron microscopy to evaluate the liposomes’ physicochemical and technological properties with some limitations mainly due to the deformation and the increased size observed. In contrast to our results, where we found a higher size for both DMPC and DSPC liposomes using AFM imaging compared with the size observed by DLS, other studies reported that AFM could be an effective tool to measure the size of the liposomes, offering comparable results with the DLS analysis.^54,55^

### EE Determination of Geraniol

Following the physicochemical characterization and stability tests of the liposomes during storage, the EE of geraniol in DMPC and DSPC lipids was analyzed separately. Overall, encapsulation is a practical and efficient processing method to protect the entrapped geraniol, whereas it supports a controlled and sustained release to inhibit the virulence factors of *S. typhimurium*, avoiding its colonization in the pig GIT.

Both DMPC- and DSPC-loaded liposomes revealed a high EE of geraniol. The EE of geraniol in the liposomes formed using the DSPC lipids was 79.33%, and when DMPC lipids were used, a higher EE efficiency of 92.53% was observed. In accordance with our results, Mohammed et al. showed that the lipid composition of the DSPC lipids due to their longer chain length can result in higher EE.^56^ Numerous types of lipids have been used to solubilize and encapsulate many kinds of hydrophobic extracts and natural compounds, including atenolol and quinine,^24^ curcumin,^46^ clove EO and its main component, eugenol,^57,58^ and cocoa extract.^59^ The EE of the compounds can vary depending on the method used to prepare the liposomes. Microfluidics has been proven to offer more stable liposomal formulations with high EE percentages in agreement with our results.^30^

### Geraniol Release from Liposomes

**Figure 6:**
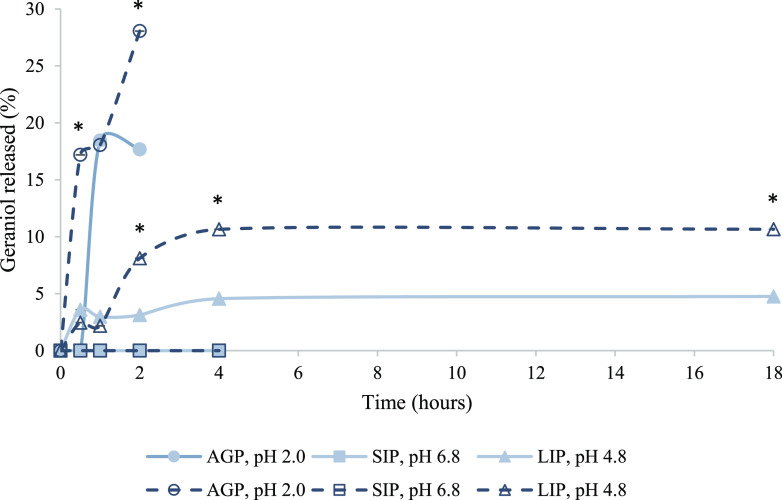
Total release rate (%) of encapsulated geraniol in simulated GIT for liposomal formulations with 14:0 PC (DMPC) (closed symbols, solid lines) and 18:0 PC (DSPC) lipids (open symbols, dashed lines). Values are presented as mean ± SD; *n* = 3. Asterisks (*) indicate the significant difference of the release profile of encapsulated geraniol from DSPC liposomes compared with the DMPC (*P* < 0.05).

To explore the release behavior of the encapsulated geraniol, the liposomes were added separately in a buffer solution with a pH value of 2.0, 6.8, and 4.8 for 2, 4, and 18 h at 39 °C simulating the pig AGP, SIP, and LIP, respectively. In Figure 6, the in vitro release profiles are presented as the percentage of geraniol released under each stage of the pig simulated GIT.

The formulation of DSPC-loaded liposomes in AGP revealed an initial burst release of geraniol at 17.19 and 18.05% after 30 min and 1 h, respectively, while the highest release of 28.06% was observed after 2 h (Figure 6). On the contrary, geraniol released from the DMPC liposomes was significantly lower and equal with 17.67% after 2 h in the same GIT phase (Figure 6, *P* < 0.05). However, geraniol was not released from both liposomal formulations under the simulated SIP (pH 6.8). The high stability under these conditions is comparable with the results of Leyva-Jiménez et al.,^60,61^ who reported that the physical stability of the liposomes remained unchanged at neutral pH 7.0 with high ionic strength, simulating the gastrointestinal fluids. When indigestible components such as EOs, flavor oils, and mineral oils are encapsulated in the lipid’s core, the formulated nanostructures such as the liposomes used in the current study may remain stable while passing the small intestine phase.^62^ Finally, the presence of free geraniol after 2 and 4 h in the LIP remained significantly higher for the DSPC formulations with 8.11 and 10.65% compared to 3.12 and 4.56% released from DMPC liposomes, followed by a sustained release profile up to 18 h (Figure 6, *P* < 0.05). In this study, we evaluated the release rate of encapsulated geraniol in each stage of the simulated pig GIT separately. Even though the liposomes performed well by controlling the immediate release of geraniol in the AGP, showing no release in the SIP and a sustained release in the LIP, it would be beneficial for the liposomes to be optimized further in future studies to increase the release rates in the LIP and achieve a more targeted delivery. Although in vitro digestion models can provide useful information, they do have limitations.^21^ There are additional factors that can influence the release of geraniol from liposomes in the animal gut. The presence of enzymes and bile salts produced during digestion can influence liposome stability and, thus, the in vivo drug release.^63,64^ Also, enzymes present during digestion (e.g., from the pancreas and gut microbiota) could also influence drug release from liposomes.^65^ Moreover, the composition of the feed given to the animal also plays a role, as liposomes may be exposed to various types of components that could interact with the lipid membrane and influence geraniol release.^66^

Therefore, in vivo release will be explored in a future study to better assess the effectiveness of geraniol.^24^ Overall, our data suggest that the DSPC liposomes revealed a faster and higher release of the encapsulated geraniol when compared with the DMPC formulations. As the drug release is one of the main endogenous factors influencing bioavailability, geraniol encapsulation in DSPC lipids could potentially lead to better bioavailability and a higher likelihood of exerting its antivirulence effects against *S. typhimurium*.

### Effect of Free and Encapsulated Geraniol on the Viability of IPEC-J2 Porcine Gut Epithelial Cells

**Figure 7:**
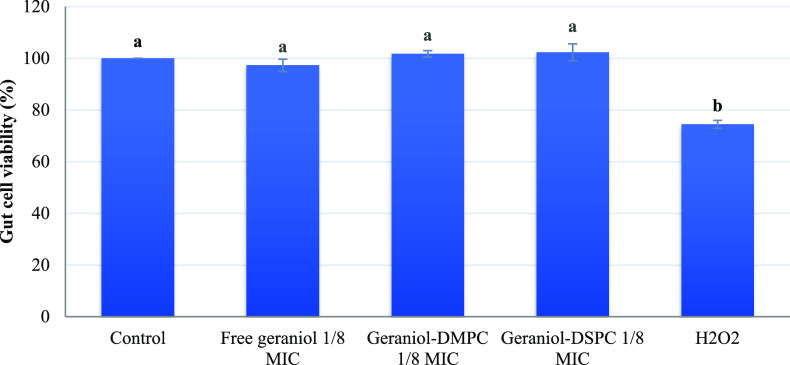
Effect of free and encapsulated geraniol in DMPC and DSPC liposomes on the viability of IPEC-J2 porcine epithelial cell line. H_2_O_2_ was used as the control. The bars represent the means ± SD; *n* = 3. Different lowercase letters indicate significant differences among treatments (*P* < 0.05).

Despite the desirable antivirulence effect of geraniol observed above, when considering the viability of the porcine cells, the potential toxicity of free and encapsulated geraniol must be addressed. Hence, the possible toxicity of free and encapsulated geraniol on the viability of IPEC-J2 porcine epithelial cells is presented in Figure 7.

The IPEC-J2 cell line has been widely used for mimicking the porcine and human gut, and in that view, this cell line is a helpful first step in screening potential diet additives prior to in vivo animal feeding.^18^ When IPEC-J2 cells were exposed to hydrogen peroxide (H_2_O_2_), a significantly lower cell viability of 74.42% was observed in comparison with the free or encapsulated geraniol (Figure 7, *P* < 0.05). Our results showed that a sub-MIC (1/8 MIC) of free or encapsulated geraniol in both DMPC and DSPC lipids had no significant adverse effect on IPEC-J2 viability compared with the control (Figure 7, *P* > 0.05). This is the first study investigating the potential cytotoxic effect of free and encapsulated geraniol on the IPEC-J2 porcine gut epithelial cells. Other studies showed that natural compounds such as carvacrol can have cytotoxic effects on gut epithelial cells^27^ and other cell lines,^67,68^ suggesting a dose-dependent cytotoxic effect. At present, there is an increased interest in the successful delivery of natural compounds in the GIT to increase their bioavailability and determine their toxicity on epithelial cells at the target site. Silva et al.^69^ reported that single (oil-in-water)- and multilayer nanoemulsions improved the bioavailability of curcumin in the GIT, but both showed a toxicity for Caco-2 cells due to the use of sodium dodecyl sulfate as a surfactant. Our results showed that encapsulated geraniol could potentially be used as an antivirulence compound targeting specific virulence factors to reduce the colonization of *S. typhimurium* without inhibiting the growth of the bacterial cells or causing cytotoxic effects to pig gut epithelial cells. Thus, the control strategy developed could potentially help increase food safety by reducing the presence of *Salmonella* in pork production. However, further in vivo studies to determine the bioavailability and biological fate of geraniol and release during digestion are necessary to prove the usefulness of this strategy conclusively.

## Abbreviations Used

AFM: atomic force microscopy
AGP: acid-gastric phase
ATR: attenuated total reflection
DLS: dynamic light scattering
DMEM: Dulbecco’s modified Eagle’s medium
DMPC: 1,2-dimyristoyl-*sn*-glycero-3-phosphocholine
DSPC: 1,2-dimyristoyl-*sn*-glycero-3-phosphocholine
EE: encapsulation efficiency
EFSA: European Food Safety Authority
EOs: essential oils
EU: European Union
FRR: flow rate ratio
FTIR: Fourier transform infrared spectroscopy
GI: gastrointestinal
GIT: gastrointestinal tract
GRAS: Generally Recognized As Safe
IPEC-J2: intestinal porcine epithelial cells
LIP: large intestine phase
MBC: minimum bactericidal concentration
MHB: Mueller–Hinton broth
MIC: minimum inhibitory concentration
MTT: 3-[4,5-dimethylthiazol-2-yl]-2,5-diphenyl tetrazolium bromide
OD: optical density
PBS: phosphate-buffered saline
PDI,: polydispersity index
SD: standard deviation
SIP: small intestine
sub-MIC: subinhibitory concentration
TFR: total flow ratio
TSA: tryptone soy agar
PC: phosphatidylcholine
USA: United States of America
